# Biochemical Characterization and Polyester-Binding/Degrading Capability of Two Cutinases from *Aspergillus fumigatus*

**DOI:** 10.3390/microorganisms13051121

**Published:** 2025-05-13

**Authors:** Haizhen Wang, Tianrui Zhang, Kaixiang Chen, Liangkun Long, Shaojun Ding

**Affiliations:** 1 National Key Laboratory for the Development and Utilization of Forest Food Resources, Nanjing Forestry University, Nanjing 210037, China; haizhen@njfu.edu.cn (H.W.); longlk602@njfu.edu.cn (L.L.); 2 Co-Innovation Center for Efficient Processing and Utilization of Forest Resources, College of Chemical Engineering, Nanjing Forestry University, Nanjing 210037, China

**Keywords:** *Aspergillus fumigatus*, cutinase, polyester adsorption/biodegradation

## Abstract

Two recombinant cutinases, *Af*CutA and *Af*CutB, derived from *Aspergillus fumigatus,* were heterologously expressed in *Pichia pastoris* and systematically characterized for their biochemical properties and polyester-degrading capabilities. *Af*CutA demonstrated superior catalytic performance compared with *Af*CutB, displaying higher optimal pH (8.0–9.0 vs. 7.0–8.0), higher optimal temperature (60 °C vs. 50 °C), and greater thermostability. *Af*CutA exhibited increased hydrolytic activity toward p-nitrophenyl esters (C4–C16) and synthetic polyesters. Additionally, *Af*CutA released approximately 3.2-fold more acetic acid from polyvinyl acetate (PVAc) hydrolysis than *Af*CutB. Quartz crystal microbalance with dissipation monitoring (QCM-D) revealed rapid adsorption of both enzymes onto polyester films. However, their adsorption capacity on poly (ε-caprolactone) (PCL) films was significantly higher than on polybutylene succinate (PBS) films, and was influenced by pH. Comparative modeling of catalytic domains identified distinct structural differences between the two cutinases. *Af*CutA possesses a shallower substrate-binding cleft, fewer acidic residues, and more extensive hydrophobic regions around the active site, potentially explaining its enhanced interfacial activation and catalytic efficiency toward synthetic polyester substrates. The notably superior performance of *Af*CutA suggests its potential as a biocatalyst in industrial applications, particularly in polyester waste bioremediation and sustainable polymer processing.

## 1. Introduction

Cutinases (E.C. 3.1.1.74) are serine esterases belonging to the α/β hydrolase superfamily. These enzymes catalyze the hydrolysis of ester linkages in natural polyesters such as cutin and suberin, essential structural components of plant apoplastic barriers [1,2,3]. They are hypothesized to aid fungal pathogen invasion by compromising plant surface integrity through polyester degradation [4]. Beyond their native substrates, cutinases exhibit substrate promiscuity, hydrolyzing a wide range of low-molecular-weight soluble esters, short- and long-chain triacylglycerols, and various synthetic polyesters (e.g., polybutylene succinate (PBS), polyethylene terephthalate (PET), poly(ε-caprolactone) (PCL)) [2,3]. This versatile catalytic capacity positions cutinases as promising biocatalysts for sustainable bioremediation and polymer upcycling [2,3,5,6,7]. Since the initial discovery of cutinase in *Fusarium solani pisi* [8], numerous cutinases have been characterized from hemibiotrophic, biotrophic, and saprotrophic fungi across diverse ecotypes [9,10,11,12,13,14,15,16,17].

Although fungal cutinases share conserved structural motifs and catalytic triads (Ser-His-Asp/Glu), significant functional divergence exists concerning substrate specificity, catalytic efficiency, optimal pH and temperature ranges, and thermostability [18]. Genomic analyses indicate that many fungal species possess multiple putative cutinase genes (CAZy database CE5 family; https://www.cazy.org/CE5.html, accessed on 1 April 2025), suggesting evolutionary specialization via gene duplication and neofunctionalization. However, systematic comparative studies on the enzymatic properties and application potentials of cutinase isoforms remain limited to a few fungal species [19,20,21].

The saprotrophic fungus *Aspergillus fumigatus* thrives in high-temperature environments (>50 °C), such as compost systems, and demonstrates a remarkable ability to degrade both synthetic polymers (e.g., PCL) and bio-based polyesters (e.g., PHB), as well as natural polysaccharides (cellulose, hemicellulose, and starch) [22]. Many polymer-degrading enzymes, including lipases, esterases, and glycoside hydrolases, have recently been identified from *A. fumigatus* [23,24,25,26]. The genome of *A. fumigatus* Af293 contains four putative cutinase genes, one of which (GenBank: XP_755273) has been functionally characterized. This enzyme exhibits higher thermostability and broader biotechnological applications in biosynthesis and polyester degradation compared with the canonical *Fusarium solani* cutinase [26]. To expand the functional catalog of *A. fumigatus* cutinases, we heterologously expressed two previously uncharacterized isoforms in *Pichia pastoris* and systematically evaluated their biochemical properties, substrate-binding dynamics, and polyester degradation efficiency. This comparative analysis elucidates the functional divergence and potential industrial applications among the cutinase isoforms from this fungus.

## 2. Materials and Methods

### 2.1. Expression and Purification of Cutinases

Three putative cutinase genes (*Afcut*A, GenBank accession No. XP_746507.1; *Afcut*B, GenBank accession No. XP_751420.1; and *Afcut*C, GenBank accession No. XP_755775.1) from *A. fumigatus* were synthesized by Sangon Biotech (Shanghai, China). Codons were optimized according to the preferred usage in *P. pastoris*. Two restriction sites (*Eco*RI and *XbaI)* were introduced at the 5′ and 3′ ends, respectively. A C-terminal 6 × His tag was fused to each gene to facilitate affinity purification. The resulting fragments were cloned into the *Eco*RI/*Xba*I sites of the pPICZαA expression vector (Invitrogen, Waltham, MA, USA), containing the *AOX*1 promoter for methanol-inducible expression and α-factor secretion signal for extracellular protein targeting. The final constructs (*pPICZαA-AfCuts*) were verified by sequencing.

The pPICZαA-*Af*Cuts plasmids were linearized with *Sac*I (New England Biolabs, Ipswich, MA, USA) and electroporated into *P. pastoris* KM71H competent cells using a Bio-Rad Gene Pulser Xcell™ system (Hercules, CA, USA) under the following conditions: 1.5 kV, 25 μF, and 200 Ω. Immediately after electroporation, cells were resuspended in 1 mL ice-cold 1 M sorbitol and incubated at 28 °C for 2 h. Transformed cells were spread onto YPDZ agar plates (1% yeast extract, 2% peptone, 2% dextrose, 2% agar) containing 100 μg/mL Zeocin™ (Thermo Fisher Scientific, Waltham, MA, USA) and incubated at 28 °C for 3–5 days until colonies appeared. Single colonies were inoculated into 5 mL YPDZ liquid medium (100 μg/mL Zeocin™) and incubated at 28 °C for 16 h with shaking at 200 rpm. Cultures were diluted 1:50 (*v*/*v*) into 50 mL buffered glycerol complex medium (BMGY) and grown at 28 °C, 200 rpm, until the OD_600_ reached 2.0–6.0. Cells were harvested by centrifugation (3000× *g*, 5 min) and resuspended in 50 mL buffered methanol-complex medium (BMMY). The cultures were maintained at 28 °C, 200 rpm for 5 days, with 1% filter-sterilized methanol added every 24 h.

After induction, cultures were centrifuged at 10,000 rpm, 10 min, 4 °C. The supernatant (crude enzyme extract) was collected and filtered through a 0.22 μm pore-size membrane. Recombinant proteins with a C-terminal 6 × His tag were purified by Ni-NTA Agarose affinity chromatography (Qiagen, Hilden, Germany) [12]. A gravity flow column packed with 1 mL Ni-NTA Agarose resin was equilibrated with 3 mL Lysis Buffer (50 mM NaH_2_PO_4_, 300 mM NaCl, 10 mM imidazole, pH 8.0). After equilibration, the crude enzyme was slowly loaded onto the column. The column was washed with 8 mL Wash Buffer (50 mM NaH_2_PO_4_, 300 mM NaCl, 20 mM imidazole, pH 8.0). Target proteins were eluted by adding 500 μL aliquots of Elution Buffer (50 mM NaH_2_PO_4_, 300 mM NaCl, 250 mM imidazole, pH 8.0). Fractions with absorbance at 280 nm (A_280_ > 1.5) were pooled as purified enzymes. The pooled eluates were dialyzed against 50 mM phosphate buffer (pH 8.0) at 4 °C for 24 h. Purity and protein concentration were determined by SDS-PAGE (12% *w*/*v*) and the BCA Protein Assay Kit (Thermo Scientific Pierce, Rockford, IL, USA), respectively, using bovine serum albumin as the standard.

### 2.2. Activity Assay

Cutinase activity was measured using *p-nitrophenyl butyrate* (*pNPB*, Sigma-Aldrich, St. Louis, MO, USA) as substrate [12]. Unless otherwise specified, the assay mixture (500 µL) contained appropriately diluted enzyme (0.075 μg protein) and 50 µL of 10 mM pNPB in 0.05 M Tris-HCl buffer (pH 8.5). The reaction was incubated at 40 °C (*Af*CutA) or 30 °C (*Af*CutB) for 5 min and terminated by adding 500 µL of 5% SDS solution. Released *p*NP was quantified by measuring absorbance at 410 nm using a Spectra MAX190 Microplate Reader (Molecular Devices, Silicon Valley, CA, USA). One unit of enzyme activity was defined as the amount of enzyme required to release 1 μmol pNP per minute under these conditions.

### 2.3. Characterization of AfCutA and AfCutB

Optimal pH and temperature values were determined using the standard assay in the ranges of pH 5.0–11.0, with the phosphate sodium buffer (pH 4.0–8.0) and glycine–NaOH (pH 8–12) at a temperature of 25–50 °C, respectively. Thermal stability was evaluated by measuring residual activities after preincubation at 35–50 °C (*Af*CutA) or 25–40 °C (*Af*CutB) without substrate for 0–20 h. To determine pH stability, enzymes were incubated for 24 h at 4 °C in buffers spanning pH 4–11, followed by activity assays at their respective optimal temperatures (40 °C for *Af*CutA, 30 °C for *Af*CutB). The activity of the non-incubated enzyme was defined as 100% for calculating relative activity. Kinetic constants (V_max_, K_m_, and k_cat_) were determined at 40 °C (*Af*CutA) and 30 °C (*Af*CutB) using 0–20 mM *pNPB* for 5 min. K_m_, V_max_, and k_cat_ were calculated by fitting data to the Michaelis–Menten equation using GraphPad Prism 7 (http://www.graphpad.com/prism/, accessed on 1 April 2025). Substrate specificity was assessed by incubating enzymes with various *pNP* esters (C2, C3, C4, C5, C8, C12; Sigma-Aldrich) under standard assay conditions. The effects of metal ions and reagents on activity were evaluated by standard assays containing metal ions at 1 or 5 mM. Organic solvent effects were assessed under optimal conditions in assays containing 50% (*v*/*v*) organic solvents.

### 2.4. Binding of AfCutA and AfCutB onto PCL and PBS Films

Poly (ε-caprolactone) (PCL, viscosity-average molecular weight: 80,000 Da) was obtained from Suzhou Zhong Zicheng Plasticizing Co., Ltd. (Suzhou, China). Poly (butylene succinate) (PBS, weight-average molecular weight: 180,000 Da) was provided by Anqing Hexing Chemical Co., Ltd. (Anqing, China). Polyvinyl acetate (PVAc, weight-average molecular weight: 70,000 Da) was obtained from Jiangsu Yinyang Gumbase Materials Co., Ltd. (Jintan, China).

The adsorption/desorption behaviors of *Af*CutA and *Af*CutB on PCL and PBS films were analyzed in situ using an E4 quartz crystal microbalance with dissipation monitoring (QCM-D, Biolin Corp., Gothenburg, Sweden). PCL or PBS (0.5% *w*/*v*) was dissolved in trichloromethane and spin-coated onto a QCM gold sensor at 3000 rpm for 1 min using a spin coater (KW-4A, Shanghai Daojing Instrument Co., Ltd., Shanghai, China). The coating process was repeated three times to ensure complete coverage of the sensor surface. Film morphology and roughness were evaluated by atomic force microscopy (AFM, Dimension Edge, Bruker Co., Ltd., Berlin, Germany). The scan size was set to 5 × 5 μm^2^ and 10 × 10 μm^2^, and at least three different locations per sample were scanned.

QCM-D measurements were conducted at 5 °C to maximally suppress enzymatic activity. This approach prevented polymer degradation during adsorption, ensuring that mass changes reflected only protein adsorption. A glycine–NaOH buffer (0.05 mol L^−1^, pH 8.0–11) was injected into the chamber until the baseline was stabilized. Diluted *Af*CutA or *Af*CutB solutions were then loaded into the chamber at a flow rate of 0.1 mL/min. Once equilibrium adsorption was achieved, the buffer solution was introduced to remove the reversibly adsorbed protein. All experiments were repeated at least three times. Changes in resonance frequency (Δf) and energy dissipation (ΔD) were recorded at a fundamental resonance frequency of 5 MHz and the third, fifth, seventh, and ninth overtones. The third overtone (15 MHz) was selected for data processing. Surface adsorption on the films was calculated according to the Sauerbrey equation [27].

△m = −C△f/n,

where Δm is the change in mass, n is the overtone order, and C is the mass sensitivity constant (17.7 ng·cm^−2^·Hz^−1^ for crystals with f_0_ = 5 MHz).

### 2.5. Degradation of Polyesters and Polyvinyl Acetate

PCL or PBS films for enzymatic degradation were prepared by dissolving 1 g of polymer in 100 mL trichloromethane, followed by spontaneous solvent evaporation at room temperature in a polytetrafluoroethylene container. The resulting films were cut into approximately 0.5 cm × 1 cm pieces. To evaluate the effect of enzyme dosage, enzymatic degradation was performed in 1 mL of glycine–NaOH buffer (pH 10.0 for *Af*CutA; pH 9.0 for *Af*CutB) containing 0.1 g of polyester film and varying enzyme concentrations (20–500 μg) at optimal temperatures (40 °C for *Af*CutA, 30 °C for *Af*CutB) with shaking at 150 rpm for 20 h. For time-course experiments, reactions were carried out similarly with 400 μg of enzyme for durations up to 48 h. Control reactions without enzymes were also conducted. After incubation, films were washed three times with deionized water. Film weights before and after degradation were measured after drying at 60 °C until constant weight.

Degradation products were extracted using ethyl acetate and analyzed by gas chromatography–mass spectrometry (GC-MS, Agilent 6890 GC/5975 MS) equipped with a DB-5 column (30 m × 0.25 mm, Agilent, Santa Clara, CA, USA). The temperature gradient was: initial temperature 60 °C (3 min), increased at 10 °C min^−1^ to 350 °C, and held at 350 °C for 4 min. Helium served as the carrier gas (flow rate: 2 mL min^−1^).

PVAc degradation was assessed by quantifying released acetic acid from a PVAc–macroporous resin composite. This composite was prepared by mixing macroporous resin with 1% (g·mL^−1^) PVAc methanol solution at a ratio of 2 g of PVAc per gram of resin, incubating at 50 °C for 20 h, and removing methanol by vacuum evaporation. This procedure was repeated twice. Enzymatic reactions were conducted in 1 mL of glycine–NaOH buffer (pH 10.0 for *Af*CutA; pH 9.0 for *Af*CutB) containing 0.1 g of composite and enzyme (400 μg of each enzyme) at optimal temperatures (40 °C for *Af*CutA, 30 °C for *Af*CutB) and 150 rpm for up to 12 h. Released acetic acid was quantified by high-performance liquid chromatography (HPLC) using an Aminex HPX-87H column (Bio-Rad, Hercules, CA, USA). The mobile phase was 5 mM H_2_SO_4_ (flow rate: 0.6 mL·min^−1^). The injection volume was 10 μL, column temperature was 20 °C, and detection was at 205 nm.

### 2.6. PCL and PBS Film Morphology Analysis by Scanning Electron Microscopy (SEM)

The surface structural changes of *PCL* and *PBS* films treated with *Af*CutA or *Af*CutB were analyzed using FEI Quanta 200 environmental scanning electron microscopy (SEM, FEI Company, Hillsboro, OR, USA) with a working voltage and distance at 15 kV and 10 mm, respectively, as described previously [11].

### 2.7. Phylogenetic Analysis, Sequence Alignment, and Structure Modelling of AfCuts

To explore the evolutionary relationships among the four cutinases from *Aspergillus fumigatus Af*293 and other fungal cutinases, 12 characterized fungal cutinases from the CE5 family (CAZy database, https://www.cazy.org/CE5.html, accessed on 1 April 2025) and 43 putative cutinases from the Eurotiomycetes group were selected from GenBank using protein BLAST (BLASTP, https://blast.ncbi.nlm.nih.gov/Blast.cgi, accessed on 1 April 2025) (Table S2). A phylogenetic tree of *Af*Cuts was constructed using the maximum-likelihood method in MEGA 7.0 and enhanced using the iTOL web platform (https://itol.embl.de/, accessed on 1 April 2025).

To further investigate similarities and differences among fungal cutinases, the amino acid sequences of four *Af*Cuts and homologous sequences from *A. fumigatiaffinis* (GenBank: KAF4240109.1) and *A. oryzae* (GenBank: BAE55151.1) were aligned using MEGA 9.0 software (https://www.megasoftware.net, accessed on 1 April 2025). Alignment visualization was enhanced using ESPript 3.0 software (https://espript.ibcp.fr/ESPript/cgi-bin/ESPript.cgi, accessed on 1 April 2025).

Homology modeling of *Af*CutA and *Af*CutB was performed using SWISS-MODEL (https://swissmodel.expasy.org/, accessed on 1 April 2025) with templates from *A. fumigatiaffinis* (PDB: 8JCT) and *A. oryzae* (PDB: 3GBS), respectively. Cavity size predictions near the active sites were performed with CavityPlus (http://www.pkumdl.cn:8000/cavityplus/index.php, accessed on 1 April 2025), and the highest-scoring cavity models were selected. Structures were visualized using PyMOL software (version 2.2.0, Schrodinger, LLC, New York, NY, USA).

## 3. Results

### 3.1. Expression and Purification of AfCuts in P. pastoris KM71H

**Figure 1:**
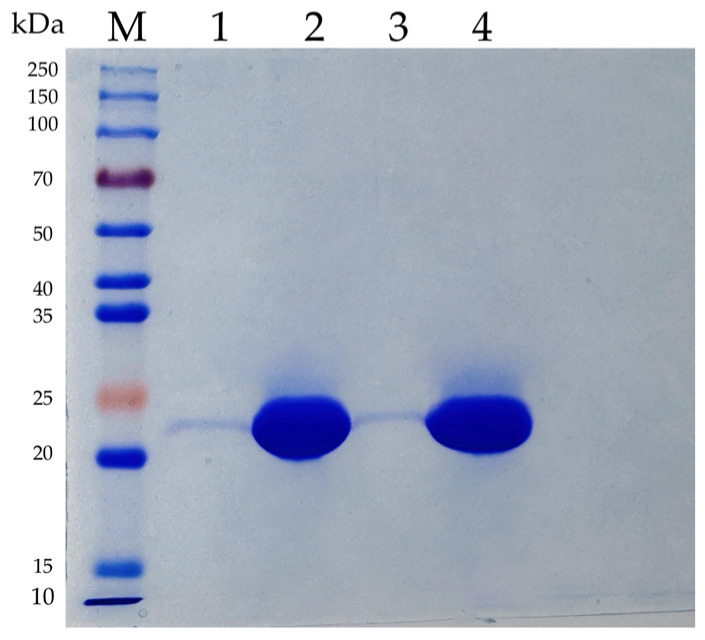
SDS-PAGE analysis of the purified recombinant *Af*CutA and *Af*CutB. Lane M, Protein Marker (EpiZyme Biomedical Technology); Lane 1 and 3, the crude enzymes of *Af*CutA and *Af*CutB, respectively; Lane 2 and 4, the purified *Af*CutA and *Af*CutB, respectively.

The codon-optimized genes encoding *Af*CutA and *Af*CutB were successfully expressed in *P. pastoris* KM71H. However, functional expression of the *Af*CutC gene was unsuccessful. His × 6-tagged *Af*CutA and *Af*CutB were purified by Ni-NTA affinity chromatography, and enzyme purity was confirmed using SDS-PAGE (Figure 1). Theoretical molecular weights of *Af*CutA and *Af*CutB were predicted as 22.48 kDa and 22.33 kDa, respectively, using ExPASy ProtParam (https://web.expasy.org/protparam/, accessed on 1 April 2025). SDS-PAGE analysis revealed experimental molecular weights of approximately 23 kDa for both *Af*CutA and *Af*CutB, closely matching the predicted values.

The effects of methanol concentration, pH, and temperature on the expression of *Af*CutA and *Af*CutB were investigated to enhance production in *P. pastoris* (Figure S1). Under optimized conditions, secreted *Af*CutA activity reached 183.8 U/mL after 6 days, representing a 14.5% increase compared with unoptimized conditions. Secreted *Af*CutB activity reached 179.8 U/mL after 6 days, representing a 26% increase compared with unoptimized conditions.

### 3.2. Effect of pH, Temperature, Metal Ions, and Organic Solvents on Enzyme Activity and Stability

**Figure 2:**
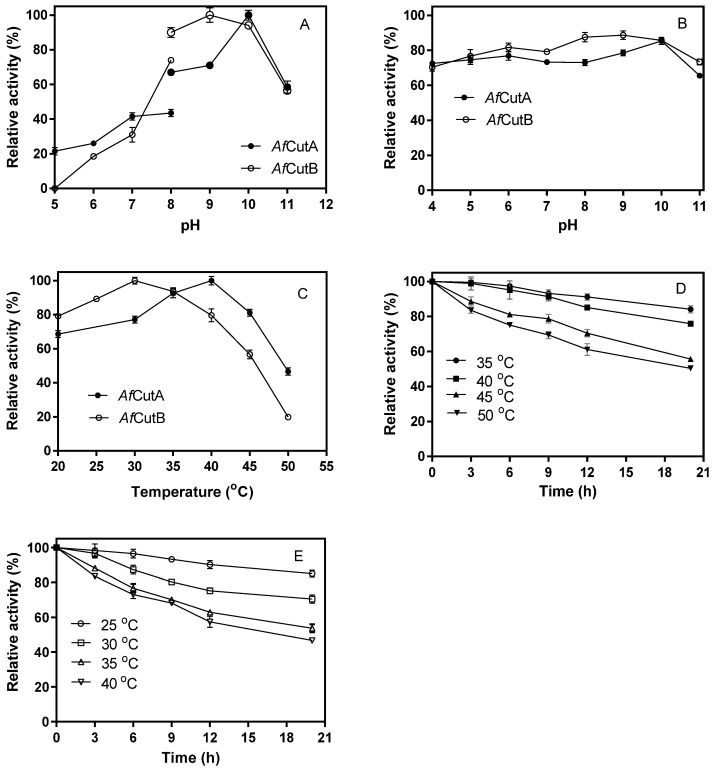
Effects of temperature and pH on the activity of *Af*CutA and *Af*CutB. (**A**) Relative enzymatic activities of *Af*CutA (40 °C) and *Af*CutB (30 °C) measured at pH 5–11. (**B**) pH stability determined after incubation at pH 4–11 (24 h, 4 °C), followed by assays at respective optimal temperatures. Activity without incubation was set as 100%. (**C**) Temperature-dependent enzyme activity measured at pH 9.0. (**D**,**E**) Residual activities after incubation at different temperatures at optimal pH values. Values represent means ± SE of triplicate measurements.

The optimal pH values for *Af*CutA and *Af*CutB were 10.0 and 9.0, respectively (Figure 2A). Both enzymes retained over 70% of their initial activity after incubation at pH 6.0–10.0 at 4 °C for 24 h (Figure 2B). Optimal temperatures were 40 °C for *Af*CutA and 30 °C for *Af*CutB (Figure 2C). *Af*CutA retained over 50% of its initial activity after incubation at 50 °C for 20 h (Figure 2D), whereas *Af*CutB retained 46% of its initial activity after incubation at 40 °C for 20 h (Figure 2E).

The effects of metal ions and other reagents on enzyme activity are shown in Table S1. Except for a slight positive effect of NH_4_^+1^ (1 and 5 mM) on *Af*CutA activity, all tested metal ions at concentrations of 1 and 5 mM slightly or moderately inhibited *Af*CutA and *Af*CutB activities. EDTA moderately inhibited both enzymes. Trichloromethane and dimethyl sulfoxide (DSMO) significantly enhanced enzyme activity, whereas other organic solvents showed slight to moderate inhibition (approximately 10–40%, Table 1).

### 3.3. Substrate Specificity and Kinetic Parameters of AfCutA and AfCutB

*Af*CutA and *Af*CutB exhibited broad substrate specificity toward p-nitrophenyl esters with acyl chain lengths ranging from C3 to C12 (Figure S2). The highest specific activities (1035.79 U/mg for *Af*CutA; 965.35 U/mg for *Af*CutB) occurred with pNPB (C4). Specific activity sharply declined when the acyl chain length differed from C4 (Figure S2). Kinetic parameters determined under optimal conditions using pNPB as substrate revealed V_max_ values of 1591 IU/g (*Af*CutA) and 1475 IU/g (*Af*CutB), and K_m_ values of 5.46 mM (*Af*CutA) and 6.29 mM (*Af*CutB) (Table 2). Compared to fungal cutinases from various sources, *Af*CutA and *Af*CutB showed relatively higher specific activities (Table 2).

### 3.4. Degradation of Polyesters and Polyvinyl Acetate

**Figure 3:**
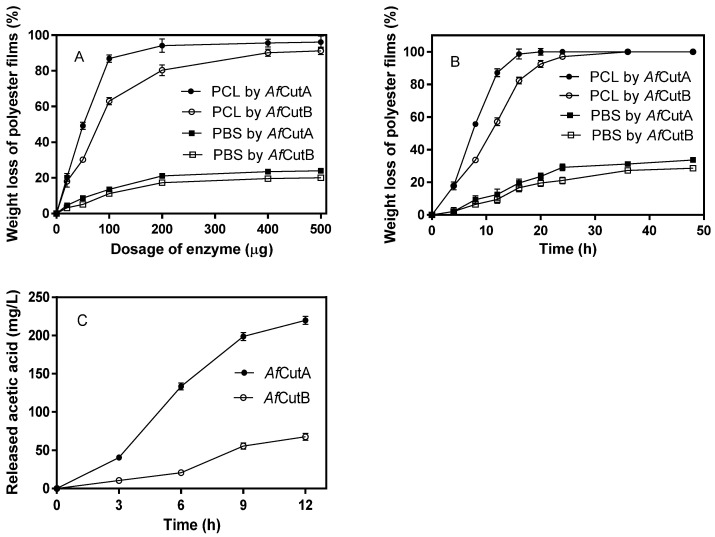
Effects of enzyme dosages on PCL and PBS film degradation by *Af*CutA or *Af*CutB (**A**). Time-course weight loss of PCL and PBS films during degradation by *Af*CutA or *Af*CutB (**B**). Released acetic acid concentration from PVAc during degradation by *Af*CutA and *Af*CutB (**C**). Values represent means ± SE of triplicate measurements.

Initially, the effects of enzyme dosages on the degradation of PCL and PBS films by *Af*CutA or *Af*CutB were evaluated (Figure 3A). Film weight loss increased rapidly as enzyme dosages rose from 0 to 100 μg, with smaller increases observed beyond this point. *Af*CutA showed higher degradation capability than *Af*CutB toward both PCL and PBS films. After a 20 h reaction with 400 μg of enzyme, *Af*CutA and *Af*CutB degraded approximately 95.6% and 90.2% of PCL film and 23.5% and 19.6% of PBS film, respectively (Figure 3A). Subsequently, time-course experiments (400 μg enzyme) were conducted (Figure 3B). *Af*CutA and *Af*CutB fully degraded PCL films within 20 h and 36 h, respectively. Approximately 29% (*Af*CutA) and 27% (*Af*CutB) of PBS films were degraded within 24 h and 36 h, respectively. The degradation rates (mg h^−1^ mg^−1^ protein) reached 54.3 ± 2.1 (*Af*CutA) and 35.3 ± 1.6 (*Af*CutB) for PCL, and 10.0 ± 1.2 (*Af*CutA) and 8.3 ± 0.3 (AfCutB) for PBS. The degradation capabilities toward PVAc were also evaluated by quantifying released acetic acid. Concentrations reached 219.8 mg/L (*Af*CutA) and 67.5 mg/L (*Af*CutB) after 12 h of hydrolysis (Figure 3C). No weight loss occurred in enzyme-free control reactions. Overall, *Af*CutA exhibited higher degradation efficiency than *Af*CutB toward PCL, PBS, and PVAc.

Degradation products from PCL and PBS films treated with *Af*CutA or *Af*CutB were analyzed by GC-MS (Figures S3 and S4). A large amount of 6-hydroxyhexanoic acid formed from PCL films, suggesting an exo-type hydrolysis mode. Trace amounts of ε-caprolactone detected may result from a back-biting mechanism [31]. 1,4-Butanediol was the sole product detected from PBS film hydrolysates.

### 3.5. SEM Analysis of AfCutA- and AfCutB-Treated PCL and PBS Films

The morphological changes of PCL and PBS films during degradation were analyzed (Figures S6 and S7). Initially, the PCL film surface appeared smooth and intact (Figure S6A). After 2 h and 4 h treatment with *Af*CutA, small cracks appeared (Figure S6B,C). After 8 h, the surface became significantly rougher with enlarged holes (Figure S6D). Similar changes were observed for *Af*CutB-treated PCL films but required longer incubation times (Figure S6E–H). PBS films degraded more slowly; minor cracks appeared after 4 h of treatment with either enzyme. Cracks gradually deepened over time, but no holes formed, even after 48 h (Figure S7). PBS films exhibited more extensive degradation by *Af*CutA compared to *Af*CutB.

### 3.6. Binding of AfCutA and AfCutB onto PCL and PBS Films

**Figure 4:**
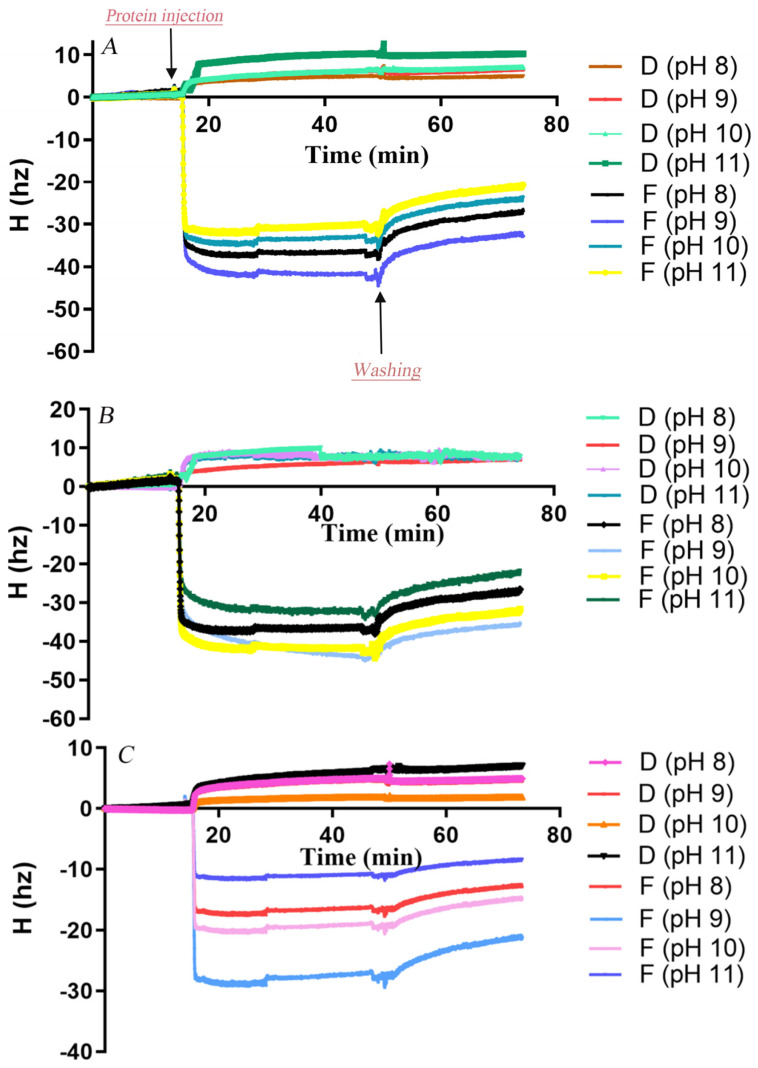

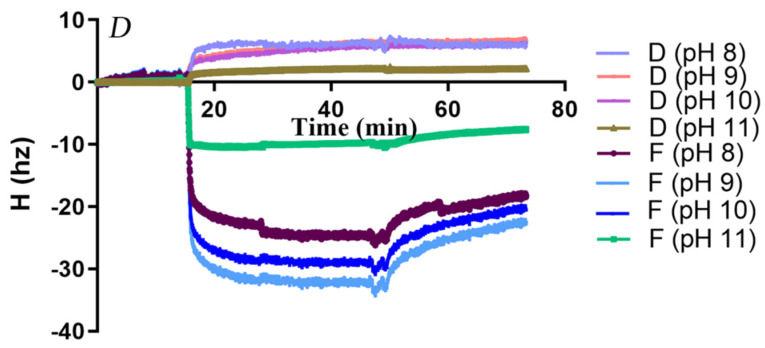
Changes in resonance frequency (Δf) and energy dissipation (ΔD) during adsorption of *Af*CutA and *Af*CutB onto polyester films. (**A**,**B**) *Af*CutA and *Af*CutB adsorption onto PCL films. (**C**,**D**) *Af*CutA and *Af*CutB adsorption onto PBS films.

Initially, the surface morphology of PCL and PBS films coated on QCM sensors was imaged by AFM (Figure S5). The sensor surfaces were fully covered by polyester, though PBS films appeared relatively rougher than PCL films. The adsorption and desorption behaviors of *Af*CutA and *Af*CutB onto PCL and PBS films at different pH values were analyzed by QCM-D. Real-time resonance frequency (Δf) and energy dissipation (ΔD) changed upon enzyme introduction and subsequent buffer washing (Figure 4). Frequency changes (Δf) reflect enzyme adsorption/desorption on polyester surfaces, whereas dissipation changes (ΔD) reflect viscoelastic interactions causing oscillation damping [32]. A rapid frequency decrease was observed upon enzyme application, indicating rapid initial adsorption of both enzymes. *Af*CutA reached adsorption equilibrium faster than *Af*CutB on both PCL and PBS films.

Adsorbed protein amounts on PCL- and PBS-coated sensors were calculated based on QCM frequency shifts (Table 3). Overall, both enzymes exhibited significantly higher adsorption onto PCL compared to PBS across pH 8–11. Maximum adsorption occurred at pH 9.0, with lower adsorption at pH values above or below this optimum. *Af*CutA was particularly sensitive to higher pH, showing a notable decrease in adsorption above pH 9. *Af*CutB displayed slightly higher adsorption than *Af*CutA on both films. During buffer washing, a greater proportion of *Af*CutB detached from PBS films compared to *Af*CutA (~30% vs. ~18%) across pH 8–11. Conversely, slightly more *Af*CutA detached from PCL films than *Af*CutB (~23% vs. ~19%), except at pH 11. Efforts to analyze adsorption/desorption behavior on PVAc films by QCM-D failed due to the inability to obtain satisfactory PVAc-coated sensors.

### 3.7. Phylogenetic Analysis and Sequence Alignment of AfCuts

**Figure 5:**
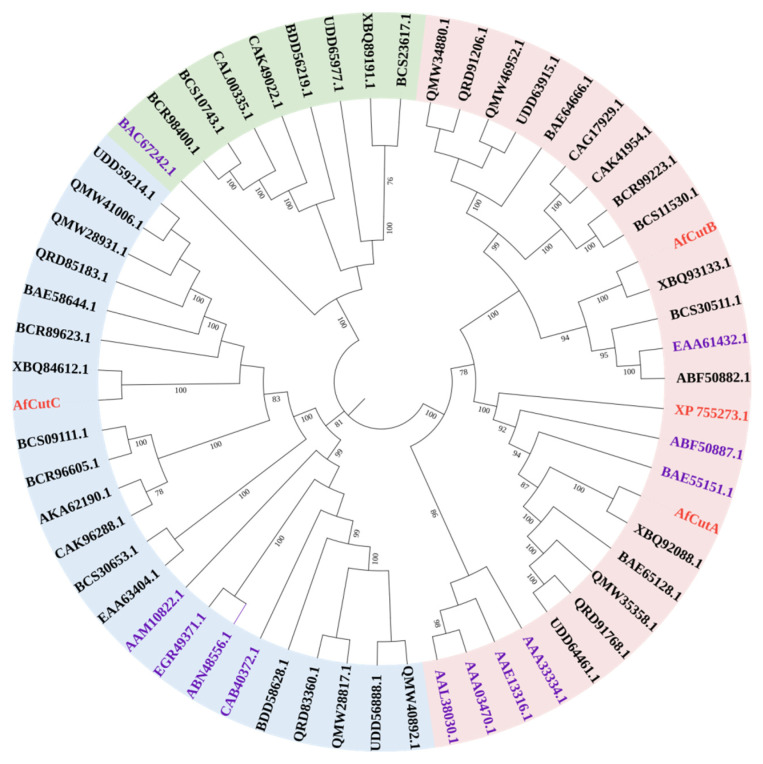
Phylogenetic analysis of fungal cutinases. TAmino acid sequences were aligned using Clustal W, and the tree was constructed using the maximum-likelihood method in MEGA 7.0. Included sequences are four cutinases from *A. fumigatus Af*293 (red), all characterized fungal cutinases of the CE5 family (purple), and putative fungal cutinases from the Eurotiomycetes group (black).

A phylogenetic tree was constructed to investigate the evolutionary relationships among four cutinases from *A. fumigatus Af*293 (Figure 5). The tree was divided into three clades. All characterized fungal cutinases from the CE5 family, including *Af*CutA, *Af*CutB, and XP 755273 from *A. fumigatus Af*293, clustered closely within one clade. *Af*CutA and *Af*CutB showed the highest sequence similarities to characterized cutinases from *A. oryzae* (BAE55151.1) and *A. nidulans* (EAA61432.1), with identities of 51.61% and 54.95%, respectively.

**Figure 6:**
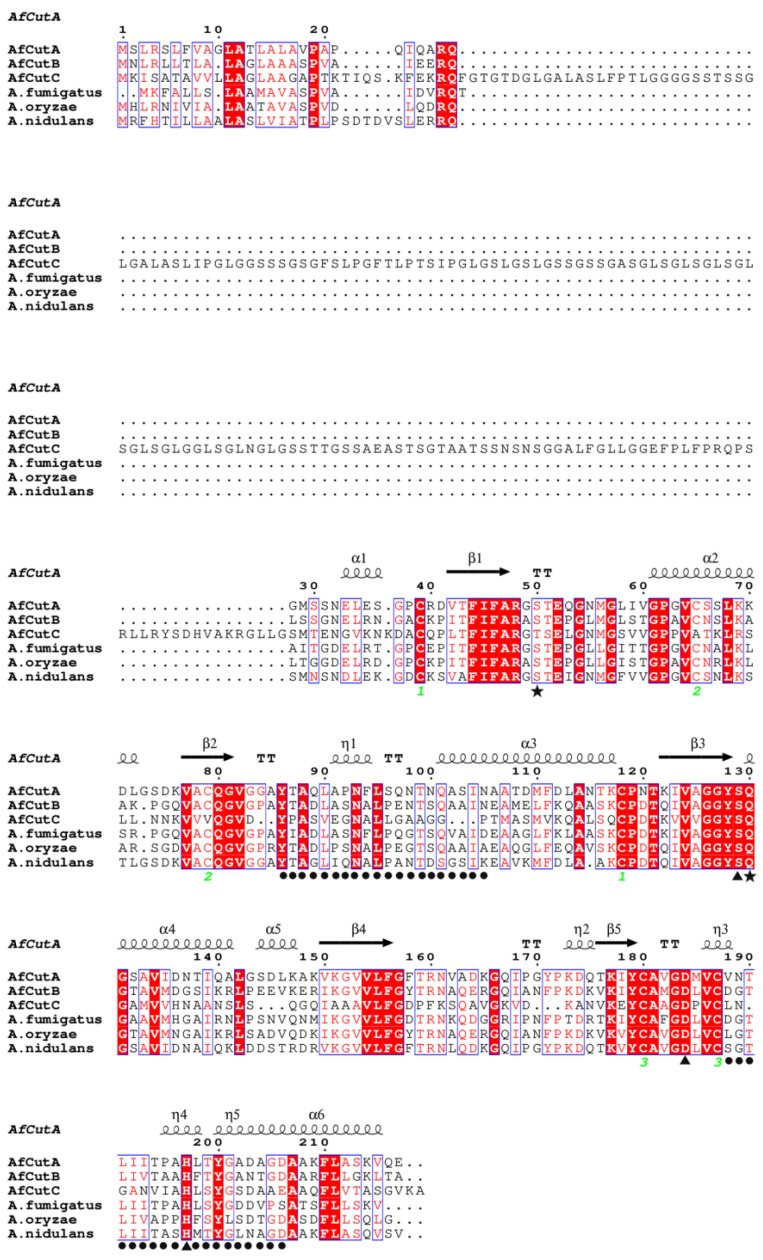
Multiple sequence alignment of *Af*CutA and *Af*CutB with other fungal cutinases. Conserved residues are shown in white on red background, semi-conserved residues in red, and non-conserved residues in black. Disulfide bonds are indicated by green numbers. *Af*CutA secondary structure elements are shown above the alignment. Catalytic triad residues (▲), oxyanion hole residues (★), and active site crown residues (●) are indicated.

Amino acid sequences of *Af*CutA, *Af*CutB, and *Af*CutC were aligned with closely related cutinases from *A. oryzae* and *A. nidulans* (Figure 6). *Af*CutA and *Af*CutB shared a 57.14% sequence similarity, whereas *Af*CutC shared only 19.38% and 20.62% similarity with *Af*CutA and *Af*CutB, respectively. Conserved motifs (GYSQG) and catalytic triad residues (SDH) were identified. *Af*CutC contains an unusually long N-terminal sequence, possibly explaining its unsuccessful expression in *P. pastoris*. *Af*CutC, like the characterized cutinase from *F. solani*, contains only two disulfide bonds [31], whereas *Af*CutA and *Af*CutB each contain three disulfide bonds. Sequence variations reflect the diversity among CE5 enzymes across different fungi or within the same fungal species.

### 3.8. Structure Modelling of AfCutA and AfCutB

**Figure 7:**
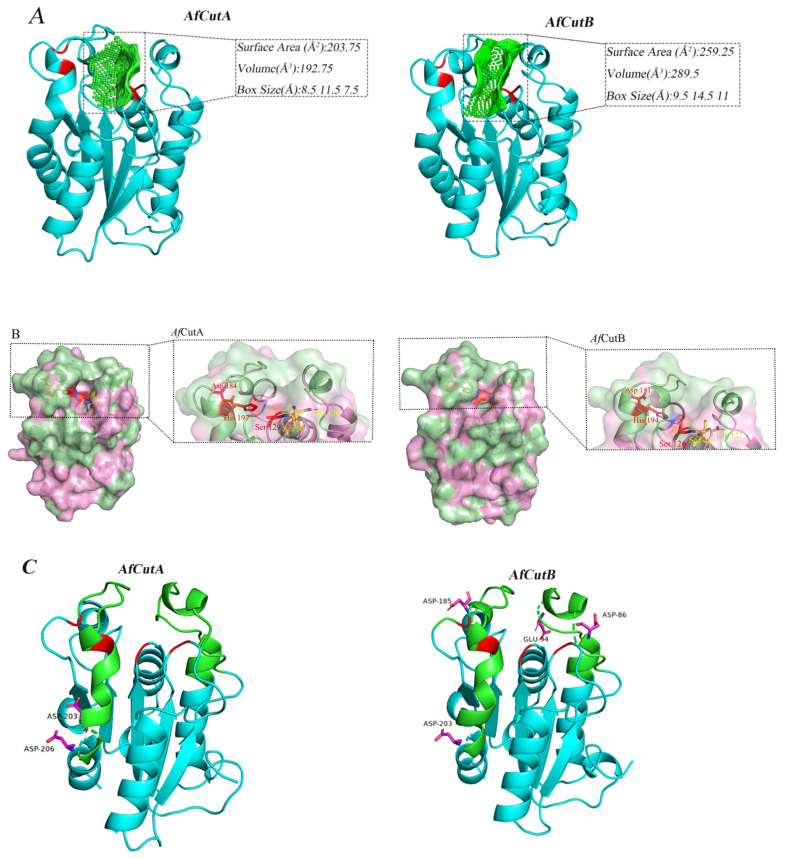
Three-dimensional structural analysis of *Af*CutA and *Af*CutB. (**A**) Substrate-binding cleft shapes and sizes of *Af*CutA and *Af*CutB. Catalytic triads, oxyanion holes, and cleft cavities are shown in red and green, respectively. (**B**) Surface models showing active sites of *Af*CutA and *Af*CutB. Surfaces are shown in pink, hydrophobic areas in pale green, catalytic triad residues in red, and oxyanion hole residues in yellow. (**C**) Analysis of acidic amino acids in the crowning area of the active sites. Crowning areas are shown in green, catalytic triads and oxyanion holes in red, and acidic residues in magenta.

The catalytic sites of both *Af*CutA and *Af*CutB were located within shallow substrate-binding clefts; however, differences existed in cavity size and depth (Figure 7A). The substrate-binding cleft of *Af*CutA was wider and shallower compared to *Af*CutB.

Typically, cutinases possess hydrophobic regions around the substrate-binding clefts, crucial for substrate recognition due to the high hydrophobicity of their natural substrates. However, the distribution of these hydrophobic areas differed between *Af*CutA and *Af*CutB (Figure 7B). In *Af*CutA, hydrophobic regions were mainly concentrated at the upper and lower areas of the substrate-binding cleft. In contrast, these areas were more dispersed in *Af*CutB, potentially leading to differences in substrate recognition efficiency [33].

Differences in biochemical properties might also result from variations in the distribution of acidic amino acids near the active sites. Structural modeling indicated that *Af*CutA contained two acidic residues (Asp203 and Asp206) in the crowning area, neither near the active site. Conversely, *Af*CutB had four acidic residues (Asp86, Glu94, Asp185, and Asp203) in the crowning area, three of which (Asp86, Glu94, and Asp185) were close to the active site (Figure 7C). Cutinases typically prefer alkaline conditions, under which acidic amino acids become negatively charged. This negative charge near the active site can result in electrostatic repulsion, thereby reducing enzymatic reactivity towards substrates [34].

## 4. Discussion

The application potential of cutinases lies primarily in their capacity to degrade synthetic polyesters [35,36]. In this study, two cutinase isoforms (*Af*CutA and *Af*CutB) from *A. fumigatus* were systematically compared, revealing significant functional divergence despite similar substrate spectra toward p-nitrophenyl esters (C4–C16). Both isoforms displayed alkaline optimal pH (*Af*CutA: pH 10.0, *Af*CutB: pH 9.0) but lower optimal temperatures (*Af*CutA: 40 °C, *Af*CutB: 30 °C) compared to previously characterized *A. fumigatus* cutinases [24]. Notably, *Af*CutA exhibited superior thermostability, retaining approximately 76% and 50% residual activity after incubation at 40 °C and 50 °C for 20 h, respectively. In contrast, AfCutB retained only 47% residual activity at 40 °C, suggesting *Af*CutA’s greater potential for higher-temperature applications.

Fungal genomes frequently encode multiple cutinase isoforms, yet comparative functional analyses remain limited to a few species. Among four cutinase genes in *A. nidulans*, only two isoforms (rCut1 and rCut2) have been thoroughly characterized, revealing distinct substrate specificities and differing PBSA degradation efficiencies [19,20]. Similarly, three cutinases from *Fusarium verticillioides* (*Fv*Cut1, *Fv*Cut2, and *Fv*Cut3) displayed variations in optimal temperature, pH, and catalytic efficiencies (k_cat_/K_m_) toward p-nitrophenyl butyrate [14]. In contrast, three cutinases from *Arxula adeninivorans* (Acut1–6hp, Acut2–6hp, and Acut3–6hp) exhibited nearly identical substrate preferences and catalytic profiles [21]. Combined with these prior findings, the current results expand understanding of fungal cutinase functional diversity and their biotechnological potential. This underscores the importance of isoform-specific characterization for industrial biocatalyst development.

The adsorption of enzymes onto polymer substrates is a critical step for enzymatic hydrolysis of insoluble polymers [37,38]. Following polyester segment hydrolysis, cutinases must desorb and re-adsorb onto fresh substrate surfaces to initiate new catalytic cycles. This adsorption-desorption dynamic critically influences polyester degradation efficiency. Unlike multi-domain hydrolytic enzymes such as cellulases or PHB depolymerases—which contain modular catalytic domains coupled with substrate-binding modules (SBMs)—cutinases generally possess only single catalytic domains [18]. Despite structural simplicity, adsorption-desorption behavior of fungal cutinases on insoluble polyester substrates are poorly understood [37,39,40,41].

In this study, QCM-D was utilized to analyze pH-dependent adsorption–desorption kinetics of *Af*CutA and *Af*CutB onto PCL and PBS films. Both enzymes exhibited rapid initial adsorption on substrates, similar to the adsorption behavior reported for *Pseudomonas cepacia* lipase on PBSL films, where saturation occurred within 5 min followed by gradual increases [42]. Adsorption capacities on PCL significantly exceeded those on PBS, correlating positively with higher PCL degradation efficiencies observed for both enzymes. These differences likely reflect distinct surface hydrophobicity and crystallinity between PCL and PBS. pH variations markedly influenced enzyme adsorption–desorption, likely by affecting enzyme surface charges. Interestingly, *Af*CutB exhibited consistent optimal pH (pH 9.0) for both p-nitrophenyl butyrate hydrolysis and substrate adsorption. Conversely, *Af*CutA displayed a mismatch between catalytic (pH 10.0) and adsorption (pH 9.0) optima. This divergence suggests distinct structure–function relationships governing catalytic activity versus substrate binding among these isoforms. It should be noted that the data obtained by QCM-D experiments at 5 °C may not really reflect the adsorption/desorption performance of enzymes in the reaction conditions.

Three-dimensional structural analyses revealed conserved overall folds in *Af*CutA and *Af*CutB despite moderate sequence similarity. However, notable differences were identified in their catalytic microenvironments. *Af*CutA possesses a shallower and more compact substrate-binding cleft compared with *Af*CutB. The active site of *Af*CutA is surrounded by denser hydrophobic regions, whereas *Af*CutB shows a more dispersed hydrophobic distribution. Moreover, *Af*CutA contains fewer acidic residues near the catalytic triad, resulting in lower local negative charge compared to *Af*CutB. These structural differences likely confer superior catalytic activity to *Af*CutA toward p-nitrophenyl esters and synthetic polyesters. The shallower cleft may enhance substrate accessibility, while concentrated hydrophobicity and reduced negative charge likely improve interfacial activation and substrate affinity [33]. Additionally, the lower density of acidic residues near the *Af*CutA active site could minimize charge repulsion against hydrophobic polyester surfaces [34]. Collectively, these findings provide insights for rational engineering of cutinases to enhance catalytic efficiency and substrate specificity.

## 5. Conclusions

In this study, two recombinant cutinases (*Af*CutA and *Af*CutB) from A. fumigatus were expressed in *P. pastoris* and comprehensively characterized for biochemical properties and polyester binding and degradation capabilities. *Af*CutA exhibited higher optimal pH, greater optimal temperature, and enhanced thermal stability compared to *Af*CutB. *Af*CutA also demonstrated higher hydrolytic activity toward p-nitrophenyl esters and synthetic polyester substrates, indicating its strong potential for biotechnological applications. To our knowledge, this study is the first to report in situ QCM-D analysis of cutinase adsorption onto polyester films. Both enzymes adsorbed more effectively onto PCL than PBS films, potentially explaining their higher degradation efficiency toward PCL. Structural modeling revealed that *Af*CutA’s shallower cleft, fewer acidic residues, and increased hydrophobic regions near the active site likely contribute to its superior catalytic performance relative to *Af*CutB.

## Figures and Tables

**Table 1:** Effect of organic solvents on *Af*CutA and *Af*CutB activities.

Organic Solvents	*Af*CutA	*Af*CutB
	Relative Activity (%)	
Control	100	100
*n*-Butanol	87.3 ± 1.3	86.1 ± 0.8
Trichloromethane	131.3 ± 1.3	121.3 ± 1.0
Cyclohexane	90.4 ± 0.5	89.3 ± 1.5
*n*-Octane	68.3 ± 1.3	61.4 ± 0.8
*n*-Hexane	70.6 ± 1.5	72.9 ± 1.7
Dimethyl sulfoxide	145.8 ± 1.3	143.7 ± 0.5
*n*-Octanol	92.2 ± 0.6	102.1 ± 2.7
Petroleum ether	88.2 ± 2.5	92.3 ± 1.5
N-N Dimethylformamide	88.3 ± 3.4	85.2 ± 2.1

The enzyme activity without organic solvents was defined as 100%. Values represent means ± SE of triplicate measurements.

**Table 2:** Comparison of biochemical properties between *Af*CutA, *Af*CutB, and other characterized fungal cutinases using various pNP esters.

Cutinase	Species	GenBank No.	MW (kDa)	Optimal pH/ Temperature (˚C)	Specific Activity (U/mg)	*K*_m_ (mM)	pNP-Ester	References
*Tt*CutA	*Thielavia terrestris*	QBX90222.1	25.3	4/50	1464	1	C4	[9]
*Mc*Cut	*Malbranchea cinnamonea*	KY5689101.1	21.9	8/45	1147.9	/	C4	[11]
*Mt*CUT	*Myceliophthora thermophila*	XP_003663956.1	23.4	8.5/30	2155	2.34	C4	[12]
*Fv*Cut1	*Fusarium verticillioides*	FVEG_12346T0	21.8	9/20	175	0.05	C4	[14]
*Fv*Cut2	*Fusarium verticillioides*	FVEG_03395	22.7	7/40	80	0.11	C4	[14]
*Fv*Cut3	*Fusarium verticillioides*	FVEG_13638	21.8	8/35	169	0.22	C4	[14]
*An*Cut2	*Aspergillus nidulans*	XP_680810.1	29	9/60	605.6	6.88	C2	[19]
*r*Cut1	*Aspergillus nidulans*	EAA62469.1	19	8/30	521	/	C5	[20]
rCut2	*Aspergillus nidulans*	EAA62121.1	29	5/30	482	/	C6	[20]
*A*cut1-6hp	*Arxula adeinivorans*	LN828946	21.6	5/20	66.1	1.6	C6	[21]
*A*cut2-6hp	*Arxula adeinivorans*	LN828947	21.6	5/30	1747	1.5	C6	[21]
*A*cut3-6hp	*Arxula adeinivorans*	LN828948	29.2	5.5/30	1251	1.9	C4	[21]
*A.fumigatus cutinase*	*Aspergillus fumigatus*	KY115674	20	8/60	1236.3	/	C4	[26]
CutAB1	*Alternaria brassicicola*	U03393.1	24	7-9/40	1057	/	C4	[28]
FSC	*Fusarium solani*	gi|2493916	24	8/50	287	1.37	C4	[29]
*Th*Cut1	*Trichoderma* *harzianum*	AJ896891	29	7.5–8	8.5	0.33	C2	[30]
*Af*CutA	*Aspergillus fumigatus*	XP_751420.1	23	10/40	1591	5.46	C4	This study
*Af*CutB	*Aspergillus fumigatus*	XP_746507.1	23	9/30	1475	6.29	C4	This study

**Table 3:** Adsorption and desorption parameters of *Af*Cuts onto PCL and PBS films at different pH conditions, measured by QCM-D.

Films	Enzyme	pH	Max Δm (ng)	Stabilized Δm′ (ng)	Wash off Δm″ (ng)
PCL	*Af*CutA	8	223.2	172.5	50.7
		9	248.5	191.5	57.0
		10	200.1	154.7	45.5
		11	170.9	132.0	38.8
	*Af*CutB	8	219.2	177.6	41.6
		9	259.8	210.5	49.3
		10	248.6	201.5	47.1
		11	188.4	134.4	54.0
PBS	*Af*CutA	8	108.0	89.9	18.0
		9	159.9	131	28.9
		10	95.9	78.6	17.3
		11	63.9	52.4	11.5
	*Af*CutB	8	145.2	105.4	39.8
		9	187.2	126.7	60.5
		10	170.1	113.5	56.7
		11	60.7	40.3	20.4

## Data Availability

The original contributions presented in this study are included in the article/Supplementary Materials. Further inquiries can be directed to the corresponding author.

## Supplementary Materials

The following supporting information can be downloaded at: https://www.mdpi.com/article/10.3390/microorganisms13051121/s1. Figure S1: (A) Effects of methanol concentration (A), pH(B), and temperature (C) on enzyme expression, and the time course of *Af*CutA (D) and *Af*CutB expression in *P. pastoris*; Figure S2: Substrate specificity of *Af*CutA and *Af*CutB towards *p*-nitrophenyl esters; Figure S3: GC-MS chromatogram of the degraded products released from PCL films by *Af*Cuts. (A) Total ion current chromatogram of samples treated by *Af*CutA. (A1-2) Mass spectrogram of degraded product 1 (6-hexanolactone) and 2 (6-hydroxycaproic acid) from samples treated by *Af*CutA, respectively. (B) Total ion current chromatogram of samples treated by *Af*CutB. (B1-2) Mass spectrogram of degraded product 1(6-hexanolactone) and 2 (6-hydroxycaproic acid) from samples treated by *Af*CutB, respectively; Figure S4: GC-MS chromatogram of the degraded products released from PBS films by *Af*Cuts. (A) Total ion current chromatogram of samples treated by *Af*CutA. (A1) Mass spectrogram of degraded product 1 (1,4-butanediol) from samples treated by *Af*CutA. (B) Total ion current chromatogram of samples treated by *Af*CutB. (B1) Mass spectrogram of degraded product 1 (1,4-butanediol) from samples treated by *Af*CutB; Figure S5: AFM scan of the PCL and PBS films coated on QCM sensors. (A,B) 10 × 10 μm^2^ and 5 × 5 μm^2^ areas of PCL film, respectively. (C,D) 10 × 10 μm^2^ and 5 × 5 μm^2^ areas of PBS film, respectively; Figure S6: Scanning electron micrographs of degraded PCL films. (A–D) After 0, 2, 4 and 8 h degradation by *Af*CutA. (E–H) After 0, 4, 8 and 12 h degradation by *Af*CutB; Figure S7: Scanning electron micrographs of degraded PBS films. (A–D) After 4, 12, 24, and 48 h degradation by *Af*CutA. (E–H) After 4, 12, 24, and 48 h degradation by *Af*CutB; Table S1: Effect of different metal ions and EDTA on the cutinase activity; Table S2: The information of the genes encoding for cutinase used in the phylogenetic tree. References [1,10,26,28,30,43,44,45,46,47,48,49] are cited in the supplementary materials.

## Abbreviations

SDS-PAGE	sodium dodecyl sulfate polyacrylamide gel electrophoresis
QCM-D	quartz crystal microbalance with dissipation monitoring
PCL	poly (ε-caprolactone)
PBS	polybutylene succinate
PVAc	polyvinyl acetate
*p*NPA	*p*-nitrophenyl acetate
*p*NPP	*p*-nitrophenyl propionate
*p*NPB	*p*-nitrophenyl butyrate
*p*NPV	*p*-nitrophenyl valerate
*p*NPC	*p*-nitrophenyl caprylate
*p*NPL	*p*-nitrophenyl laurate
AFM	an atomic force microscopy
SEM	electron microscopy
DSMO	dimethyl sulfoxide
GC-MS	gas chromatography–mass spectrometry
