# Supplementary material for: Biochemical Characterization and Polyester-Binding/Degrading Capability of Two Cutinases from Aspergillus fumigatus
Source: Microorganisms. 2025 May 13;13(5):1121. doi: 10.3390/microorganisms13051121 (PMC12114444; doi:10.3390/microorganisms13051121)
Supplement: Supplementary file 1 [file microorganisms-13-01121-s001.zip › microorganisms-3591740-supplementary.pdf]

microorganisms

Supporting Information

Biochemical Characterization and Polyester-Binding/ Degrading Capability of Two Cutinases from *Aspergillus fumigatus*

Haizhen Wang [1,2,†], Tianrui Zhang [1,2,†], Kaixiang Chen [1,2], Liangkun Long [1,2] and Shaojun Ding [1,2,*]

1 National Key Laboratory for the Development and Utilization of Forest Food Resources, Nanjing Forestry University, Nanjing 210037, China
2 Co-Innovation Center for Efficient Processing and Utilization of Forest Resources, College of Chemical Engineering, Nanjing Forestry University, Nanjing 210037, China
* Correspondence: dshaojun@hotmail.com; Tel: +86 25 85427939; Fax: +86 25 85418873
† These authors contributed equally to this work.

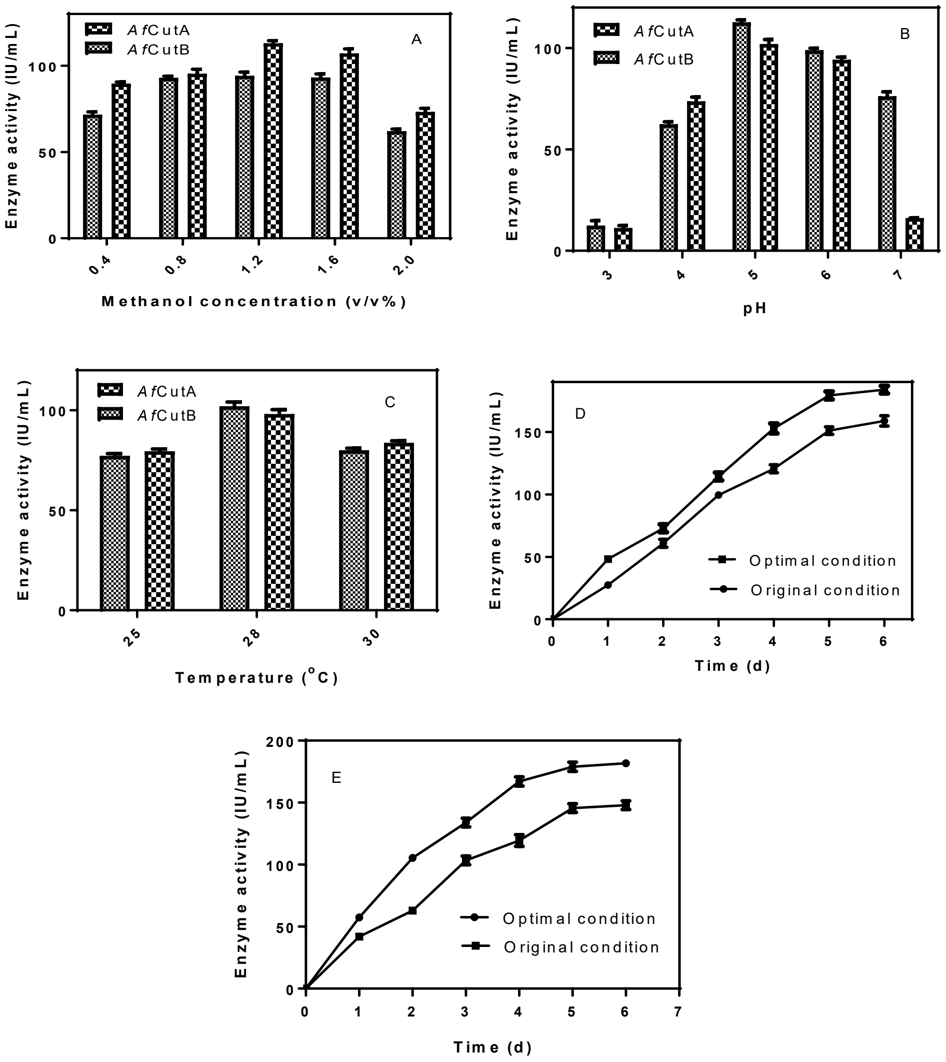

Figure S1. (**A**) Effects of methanol concentration (**A**), pH (**B**), and temperature (**C**) on enzyme expression, and the time course of *Af*CutA (**D**) and *Af*CutB expression in *P. pastoris* (**E**).

Table S1. Effect of different metal ions and EDTA on the cutinase activity.

Concentration	1 mM	5 mM	1 mM	5 mM
	***Af*CutA**		***Af*CutB**	
Mn^{2+}	88.27±1.14	81.81±2.15	77.32±2.86	77.41±4.66
NH^{4+}	105.46±1.11	103.30±4.17	84.13±2.64	74.52±11.01
Ca^{2+}	90.59±4.31	92.21±3.15	99.35±3.85	98.04±0.88
Cu^{2+}	74.59±3.51	72.16±2.41	70.09±0.27	77.45±0.47
Zn^{2+}	87.97±4.31	79.73±2.68	86.47±3.82	93.57±4.21
Fe^{3+}	84.44±4.61	83.76±1.31	89.54±5.64	74.51±0.63
Ni^{+}	100.94±4.41	85.04±5.65	85.05±1.87	78.12±5.29
Mg^{2+}	93.93±3.68	84.57±3.15	86.74±0.35	94.72±0.33
EDTA	80.55±3.24	82.21±1.88	69.40±3.26	74.74±10.51

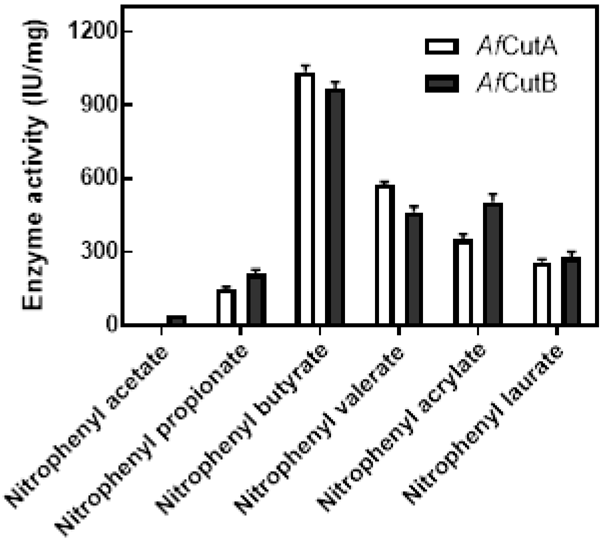

Figure S2. Substrate specificity of *Af*CutA and *Af*CutB towards *p*-nitrophenyl esters.

Table S2. The information of the genes encoding for cutinase used in the phylogenetic tree.

	Protein Name	Organism	GenBank Nos	Note
*Af*293	*Af*CutA	*Aspergillus fumigatus*	XP_746507.1	This study
	*Af*CutB	*Aspergillus fumigatus*	XP_751420.1	This study
	*Af*CutC	*Aspergillus fumigatus*	XP_755775.1	This study
CE5(Characterized)	*A. fumigatus*	*Aspergillus fumigatus*	XP_755273.1	[1]
	*Ab*CutAB1	*Alternaria brassicicola*	AAA03470.1	[2]
	*An*7541.2	*Aspergillus nidulans*	ABF50887.1	[3]
	*An*7180.2	*Aspergillus nidulans*	EAA61432.1	[3]
	*Ao*090005000029	*Aspergillus oryzae*	BAE55151.1	[4]
	Cle1	*Cryptococcus*	BAC67242.1	[5]
	*Fs*Cut1	*Fusarium solani*	AAA33334.1	[6]
	*Gc*Cut	*Glomerella cingulata*	AAL38030.1	[7]
	*Hi*Cut	*Humicola insolens*	AAE13316.1	[8]
	*Mf*Cut1	*Monilinia fructicola*	AAM10822.1	[9]

*Pbc*1	*Pyrenopeziza brassicae*	CAB40372.1	[10]
*Th*Cut1	*Trichoderma harzianum*	ABN48556.1	[11]
4psc	*Trichoderma reesei*	EGR49371.1	[12]
	Aspergillus flavus	QMW34880.1	\
	Aspergillus flavus	QRD91206.1	\
	Aspergillus flavus	QMW46952.1	\
	Aspergillus flavus	UDD63915.1	\
	Aspergillus oryzae	BAE64666.1	\
	Aspergillus niger	CAG17929.1	\
	Aspergillus niger	CAK41954.1	\
	Aspergillus luchuensis	BCR99223.1	\
	Aspergillus luchuensis	BCS11530.1	\
	Aspergillus fumigatus	XBQ93133.1	\
	Aspergillus puulaauensis	BCS30511.1	\
	Aspergillus nidulans	ABF50882.1	\
	Aspergillus fumigatus	XBQ92088.1	\
	Aspergillus oryzae	BAE65128.1	\
	Aspergillus flavus	QMW35358.1	\
	Aspergillus flavus	QRD91768.1	\
	Aspergillus flavus	UDD64461.1	\
	Aspergillus flavus	QMW40892.1	\
	Aspergillus flavus	UDD56888.1	\
	Aspergillus flavus	QMW28817.1	\
	Aspergillus flavus	QRD83360.1	\
Hypothetical protein (within the Eurotiomycetes group)	*Monascus purpureus*	BDD58628.1	\
	Aspergillus nidulans	EAA63404.1	\
	Aspergillus puulaauensis	BCS30653.1	\
	Aspergillus niger	CAK96288.1	\
	Aspergillus niger	AKA62190.1	\
	Aspergillus luchuensis	BCR96605.1	\
	Aspergillus luchuensis	BCS09111.1	\
	Aspergillus fumigatus	XBQ84612.1	\
	Aspergillus chevalieri	BCR89623.1	\
	Aspergillus oryzae	BAE58644.1	\
	Aspergillus flavus	QRD85183.1	\
	Aspergillus flavus	QMW28931.1	\
	Aspergillus flavus	QMW41006.1	\
	Aspergillus flavus	UDD59214.1	\
	Aspergillus luchuensis	BCR98400.1	\
	Aspergillus luchuensis	BCS10743.1	\
	Aspergillus niger	CAL00335.1	\
	Aspergillus niger	CAK49022.1	\
	Monascus purpureus	BDD56219.1	\
	Aspergillus flavus	UDD65977.1	\
	Aspergillus fumigatus	XBQ89191.1	\
	Aspergillus puulaauensis	BCS23617.1	\

“\”: Hypothetical proteins that have a gene accession number in the NCBI but no literature references.

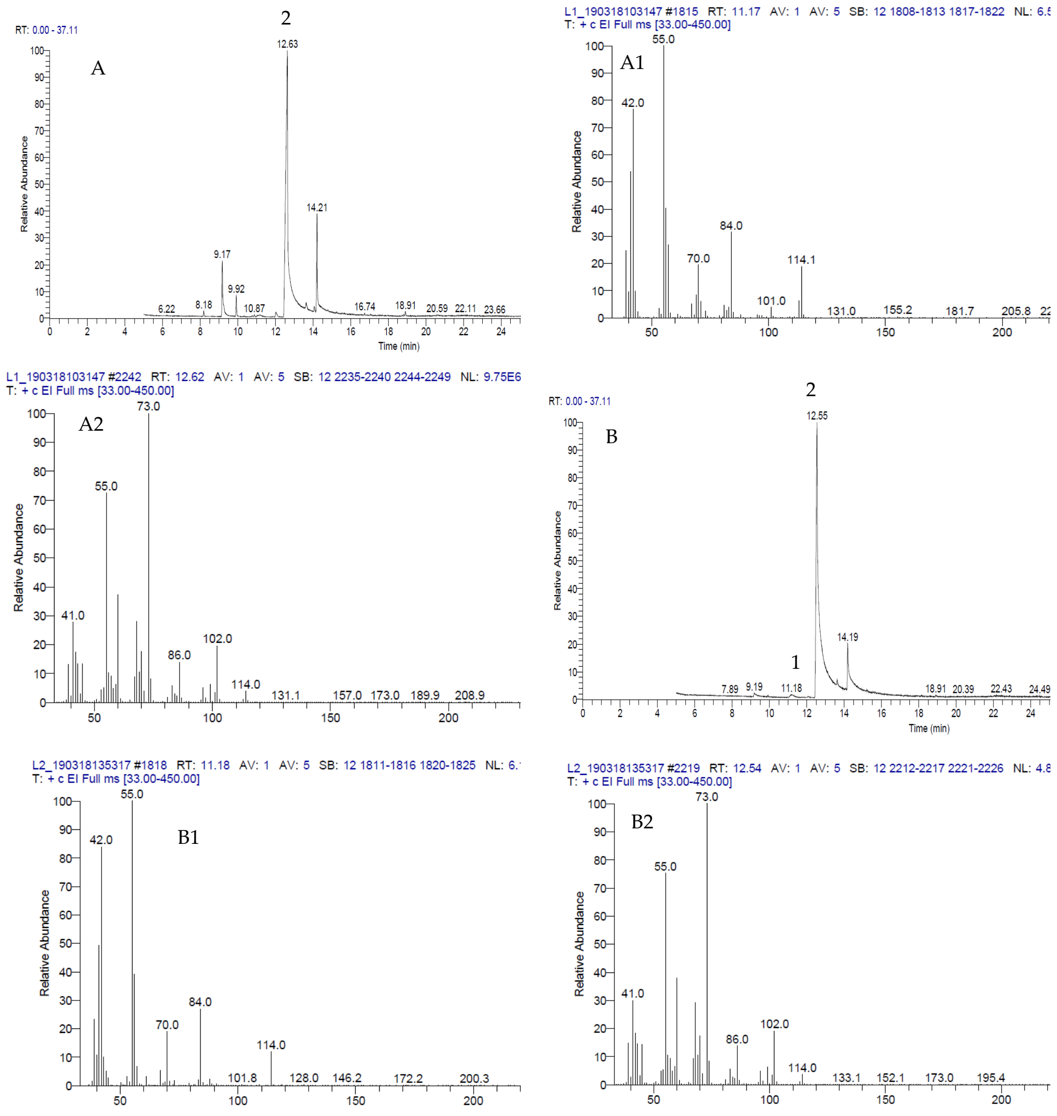

Figure S3. GC-MS chromatogram of the degraded products released from PCL films by *Af*Cuts. (**A**) Total ion current chromatogram of samples treated by *Af*CutA. (**A1**–**2**) Mass spectrogram of degraded product 1 (6-hexanolactone) and 2 (6-hydroxycaproic acid) from samples treated by *Af*CutA, respectively. (**B**) Total ion current chromatogram of samples treated by *Af*CutB. (**B1**–**2**) Mass spectrogram of degraded product 1(6-hexanolactone) and 2 (6-hydroxycaproic acid) from samples treated by *Af*CutB, respectively.

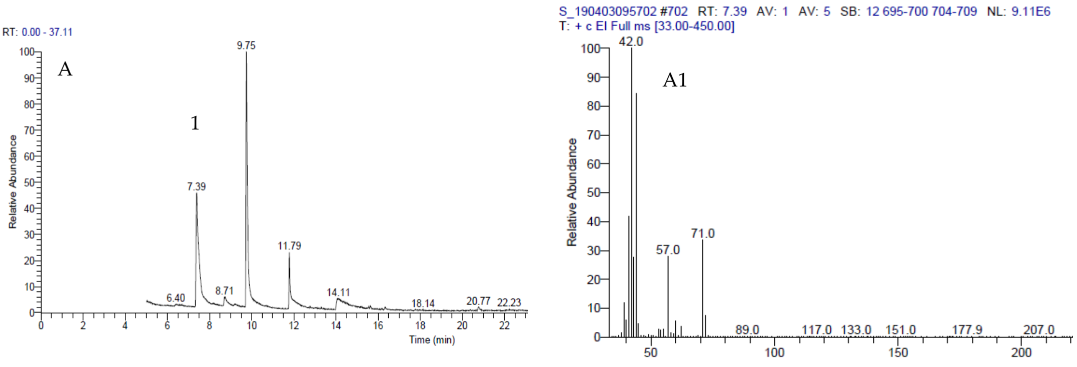

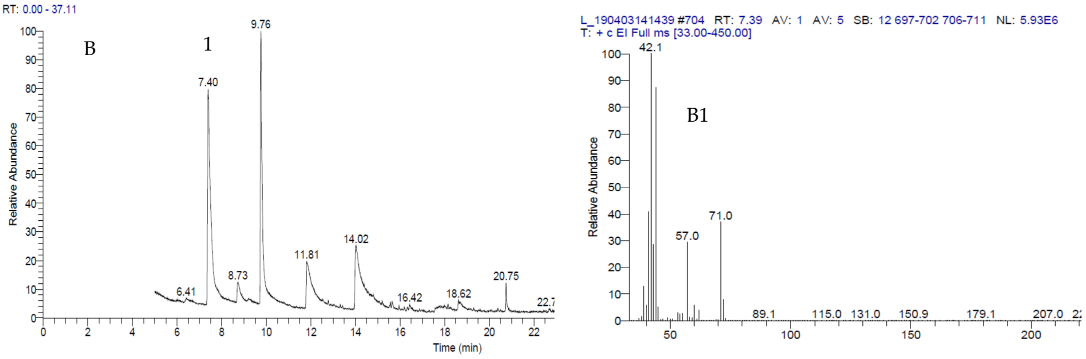

Figure S4. GC-MS chromatogram of the degraded products released from PBS films by *Af*Cuts. (**A**) Total ion current chromatogram of samples treated by *Af*CutA. (**A1**) Mass spectrogram of degraded product 1(1,4-butanediol) from samples treated by *Af*CutA. (**B**) Total ion current chromatogram of samples treated by *Af*CutB. (**B1**) Mass spectrogram of degraded product 1(1,4-butanediol) from samples treated by *Af*CutB..

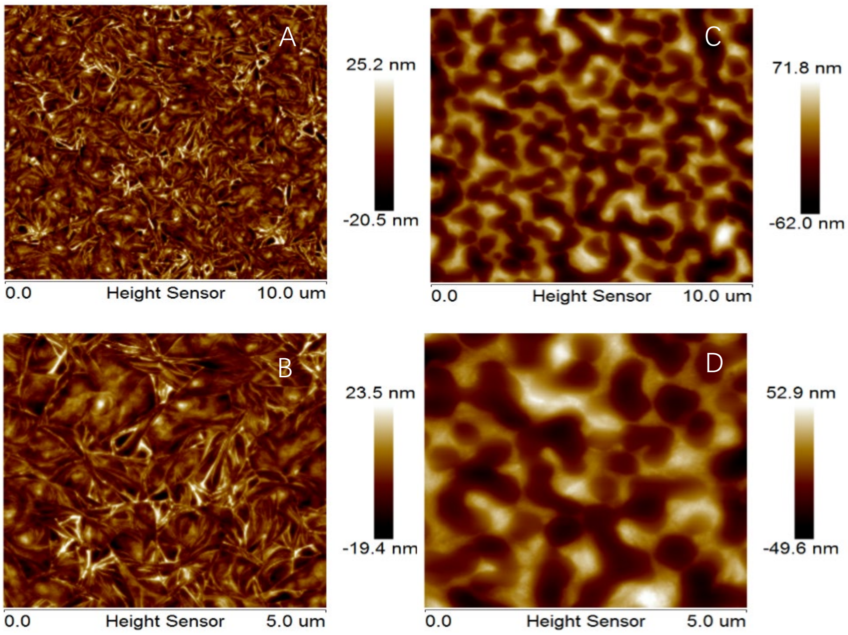

Figure S5. AFM scan of the PCL and PBS films coated on QCM sensors. (**A**–**B**) 10 × 10 μm^2 and 5 × 5 μm^2 areas of PCL film, respectively. (**C**–**D**) 10 × 10 μm^2 and 5 × 5 μm^2 areas of PBS film, respectively.

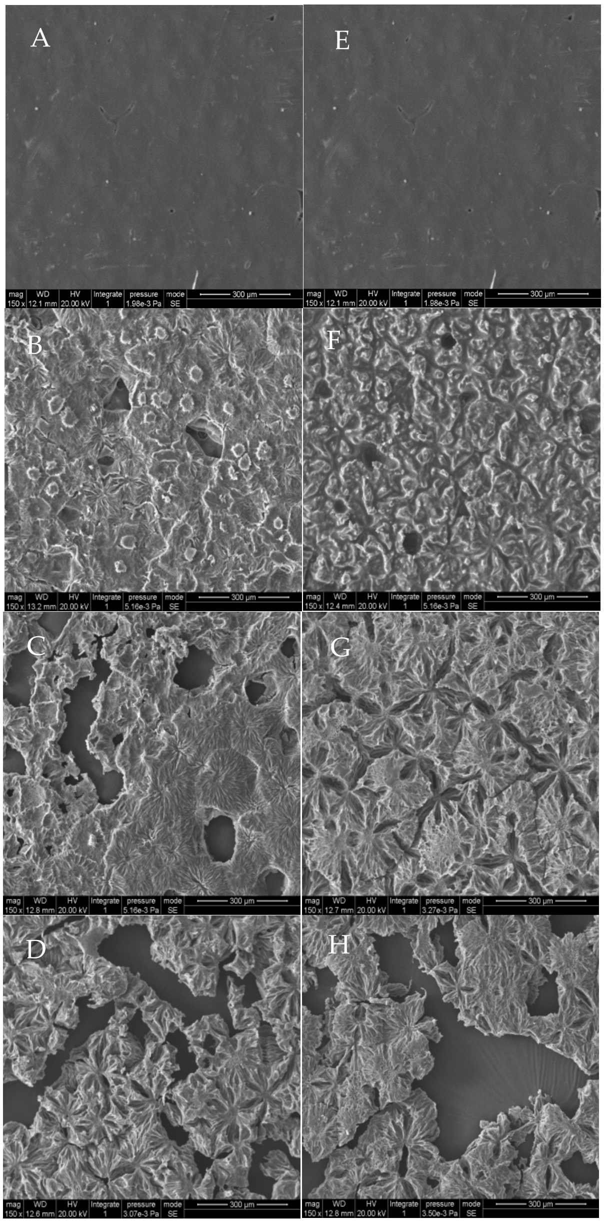

Figure S6. Scanning electron micrographs of degraded PCL films. (**A**–D) After 0, 2, 4 and 8 h degradation by *Af*CutA. (**E**–**H**) After 0, 4, 8 and 12 h degradation by *Af*CutB.

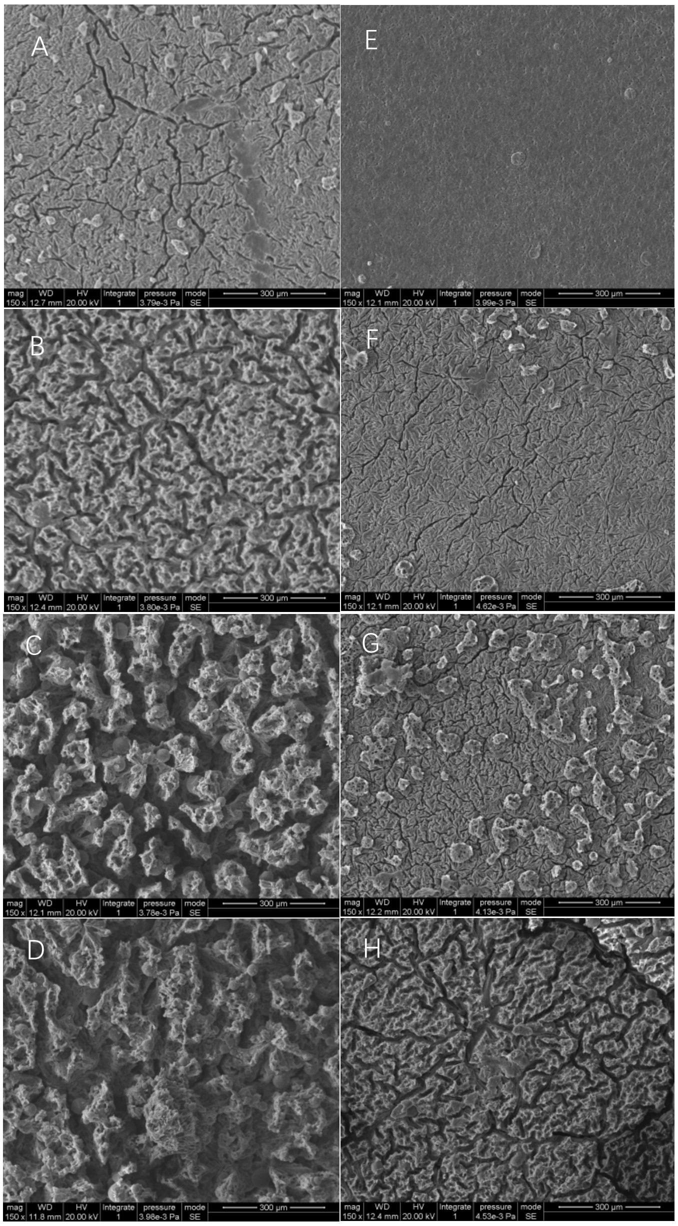

Figure S7. Scanning electron micrographs of degraded PBS films. (**A**–**D**) After 4, 12, 24, and 48 h degradation by *Af*CutA. (**E**–**H**) After 4, 12, 24, and 48 h degradation by *Af*CutB.

Supporting References

1. Ping, L.F.; Chen, X.Y.; Yuan, X.L.; Zhang, M.; Chai, Y.J.; Shan, S.D. Application and comparison in biosynthesis and biodegradation by *Fusarium solani* and *Aspergillus fumigatus*cutinases. *Int. J. Biol. Macromol*. **2017**, *104*, 1238–1245. https://doi.org/10.1016/j.ijbiomac.2017.06.118.
2. Koschorreck, K.; Liu, D.N.; Kazenwadel, C.; Schmid, R.D.; Hauer, B. Heterologous expression, characterization and site-directed mutagenesis of cutinase CUTAB1 from *Alternaria brassicicola*. *Appl. Microbiol. Biotechnol*. **2010**, 87, 991–997. https://doi.org/10.1007/s00253-010-2533-3.
3. Bauer, S.; Vasu, P.; Persson, S.; Mort, A.J.; Somerville, C.R. Development and application of a suite of polysaccharide-degrading enzymes for analyzing plant cell walls. *Proc. Natl. Acad. Sci. USA* **2006**, *103*, 11417-11422. https://doi.org/10.1073/pnas.0604632103.
4. Liu, Z.Q.; Gosser, Y.; Baker, P.J.; Ravee, Y.; Lu, Z.Y.; Alemu, G.; Li, H.G.; Butterfoss, G.L.; Kong, X.P.; Gross, R.; et al. Structural and Functional Studies of *Aspergillus oryzae* Cutinase: Enhanced Thermostability and Hydrolytic Activity of Synthetic Ester and Polyester Degradation. *J. Am. Chem. Soc.* **2009**, *131*, 15711–15716. https://doi.org/10.1021/ja9046697.
5. Kodama, Y.; Masaki, K.; Kondo, H.; Suzuki, M.; Tsuda, S.; Nagura, T.; Shimba, N.; Suzuki, E.; Iefuji, H. Crystal structure and enhanced activity of a cutinase-like enzyme from *Cryptococcus* sp strain S-2. *Proteins* **2009**, *77*, 710–717. https://doi.org/10.1002/prot.22484.

6. Martinez, C.; Geus, P.D.; Lauwereys, M.; Matthyssens, G.; Cambillau, C. Fusarium solani cutinase is a lipolytic enzyme with a catalytic serine accessible to solvent. *Nature* 1992, *356*, 615–618. https://doi.org/10.1038/356615a0.
7. Nyon, M.P.; Rice, D.W.; Berrisford, J.M.; Hounslow, A.M.; Moir, A.J.G.; Huang, H.Z.; Nathan, S.; Mahadi, N.M.; Abu Bakar, F.D.; Craven, C.J. Catalysis by *Glomerella cingulata* Cutinase Requires Conformational Cycling between the Active and Inactive States of Its Catalytic Triad. *J. Mol. Biol*. **2009**, *385*, 226-235. https://doi.org/10.1016/j.jmb.2008.10.050.
8. Kold, D.; Dauter, Z.; Laustsen, A.K.; Brzozowski, A.M.; Turkenburg, J.P.; Nielsen, A.D.; Koldso, H.; Petersen, E.; Schiott, B.; Maria, L.D.; et al. Thermodynamic and structural investigation of the specific SDS binding of *Humicola insolens* cutinase. *Protein Sci*. **2014**, *23*, 1023-1035. https://doi.org/10.1002/pro.2489.
9. Wang, G.Y.; Michailides, T.J.; Hammock, B.D.; Lee, Y.M.; Bostock, R.M. Molecular cloning, characterization, and expression of a redox-responsive cutinase from *Monilinia fructicola* (Wint.) honey. *Fungal Genet. Biol*. **2002**, *35*, 261–276. https://doi.org/10.1006/fgbi.2001.1320.
10. Li, D.H.; Ashby, A.M.; Johnstone, K. Molecular evidence that the extracellular cutinase Pbc1 is required for pathogenicity of *Pyrenopeziza brassicae* on oilseed rape. *Mol. Plant-Microbe Interact*. **2003**, *16*, 545–552. https://doi.org/10.1094/MPMI.2003.16.6.545.
11. Rubio, M.B.; Cardoza, R.E.; Hermosa, R.; Gutiérrez, S.; Monte, E. Cloning and characterization of the Thcut1 gene encoding a cutinase of *Trichoderma harzianum* T34. *Curr. Genet*. **2008**, *54*, 301–312. https://doi.org/10.1007/s00294-008-0218-6.
12. Roussel, A.; Amara, S.; Nyyssölä, A.; Mateos-Diaz, E.; Blangy, S.; Kontkanen, H.; Westerholm-Pantinen, A.; Carrière, F.; Cambillau, C. A Cutinase from *Trichoderma reesei* with a Lid-Covered Active Site and Kinetic Properties of True Lipases. *J. Mol. Biol*. **2014**, *426*, 3757–3772. https://doi.org/10.1016/j.jmb.2014.09.003.

Disclaimer/Publisher's Note: The statements, opinions and data contained in all publications are solely those of the individual author(s) and contributor(s) and not of MDPI and/or the editor(s). MDPI and/or the editor(s) disclaim responsibility for any injury to people or property resulting from any ideas, methods, instructions or products referred to in the content.
